# Screening for Pulmonary Hypertension in Systemic Sclerosis—A Primer for Cardio-Rheumatology Clinics

**DOI:** 10.3390/diagnostics11061013

**Published:** 2021-06-01

**Authors:** Adrian Giucă, Carina Mihai, Ciprian Jurcuț, Ana Maria Gheorghiu, Laura Groșeanu, Alina Dima, Adrian Săftoiu, Ioan Mircea Coman, Bogdan A. Popescu, Ruxandra Jurcuț

**Affiliations:** 1 Department of Cardiology, “Prof. Dr. C.C. Iliescu” Emergency Institute for Cardiovascular Diseases, Fundeni Street no. 258, 022328 Bucharest, Romania; adriangiuca17@gmail.com (A.G.); iocoman@yahoo.com (I.M.C.); bogdan.a.popescu@gmail.com (B.A.P.); 2 Faculty of Medicine, “Carol Davila” University of Medicine and Pharmacy, Str Dionisie Lupu nr 37, 020021 Bucharest, Romania; carmenmarinamihai@yahoo.ro (C.M.); ana.gherghe@gmail.com (A.M.G.); secretariat@spitalsfmaria.ro (L.G.); 3 Department of Rheumatology, University Hospital Zurich, University of Zurich, 8091 Zurich, Switzerland; 4 Department of Internal Medicine, “Dr. Carol Davila” Central Military Emergency University Hospital, 010825 Bucharest, Romania; cjurcut@gmail.com; 5 Department of Rheumatology and Internal Medicine, Cantacuzino Clinical Hospital, Str.Ion Movilă nr 5-7, 020475 Bucharest, Romania; 6 Department of Rheumatology, “Sf. Maria” Clinical Hospital, Bd Ion Mihalache nr 37-39, 011172 Bucharest, Romania; 7 Department of Rheumatology, Colentina Clinical Hospital, Sos Stefan cel Mare nr 19-21, 020125 Bucharest, Romania; secretariat@spitalulcolentina.ro; 8 Department of Internal Medicine, Craiova University of Medicine and Pharmacy, Str Petru Rares nr 2, 200349 Craiova, Romania; adriansaftoiu@gmail.com

**Keywords:** systemic sclerosis, pulmonary hypertension, screening, cardio-rheumatology

## Abstract

Systemic sclerosis (SSc) is a rare disease, with unfavorable clinical course and prognosis, characterized by progressive multisystemic involvement. SSc associated pulmonary hypertension (SSc-PAH) and interstitial lung disease (ILD) are the most important factors for morbi-mortality in these patients, being responsible for more than 60% of total deaths. Though pulmonary arterial hypertension (PAH) is the dominant subtype seen in SSc, PH secondary to ILD, left-heart pathology, and pulmonary veno-occlusive disease (PVOD) are also possible occurrences. Initial evaluation of a SSc case is complex and should be performed with a multidisciplinary approach. Early detection of SSc-PAH is imperative, given the fact that new and effective medications are available and early treatment was shown to improve outcomes. Therefore, screening algorithms must be used adequately and in a cost-effective manner. Sensitivity and negative predictive value (NPV) are the most important performance measures in a screening test. Several algorithms were developed in the last decade (e.g., DETECT and ASIG) and demonstrated higher efficiency when compared to older algorithms. The present manuscript details the risk factors for SSc-PAH and includes a critical description of current detection algorithms, as a primer for clinicians working in the field of cardio-rheumatology.

## 1. Introduction

Systemic sclerosis (SSc) is a rare disease, with unfavorable clinical course and prognosis, characterized by progressive multisystemic involvement, which in the end causes disability and death [1]. The reported prevalence of SSc is 7.2–33.9 and 13.5–44.3 per 100,000 individuals in Europe and North America, respectively [2]. This leads to an estimated case load of 9–19/million in the United States, with close to 100,000 patients to date [1]. There is female gender predominance (4.6/1), and although this condition can be diagnosed at any age, the highest incidence is between 30 and 50 years [1].

From a pathophysiological viewpoint, the triggering mechanism is unknown, yet there is genetic predisposition and probably exposure to environmental factors: infectious, toxic, drugs, occupational factors, etc., triggering autoimmunity and microvascular changes [1]. The natural history of SSc has two different stages: the initial phase is dominated by micro-vasculopathy and sometimes also inflammatory manifestations, while the late stage, which in some patients never develops, is mainly characterized by fibrosis of the skin and internal organs [1]. Data from EUSTAR and a cohort of French death certificates of SSc patients proved that primary heart disease is the main cause of death in SSc, explaining 30% of SSc deaths, followed by respiratory causes related to interstitial lung disease (ILD) [3]. Therefore, SSc associated pulmonary arterial hypertension (PAH) (SSc-PAH) and ILD are the most important factors for morbi-mortality in these patients, being responsible for more than 60% of total deaths [4,5].

Two different subsets of SSc are described, according to the extent of skin fibrosis: limited cutaneous (lcSSc) or diffuse cutaneous (dcSSc), with certain immunologic profiles and associated internal organ involvement [1]. Patients with lcSSc have immunology characterized by anti-centromere antibodies (ACA), and more frequently develop PAH as a late complication [1]. In contrast, patients with dcSSc usually have a more severe disease course and are associated significantly higher rates of ILD [1]. Anti-topoisomerase 1 (Scl-70) and anti-RNA polymerase III antibodies are most frequently found in this subset of patients [1].

Pulmonary hypertension (PH) encompasses a group of severe clinical entities in which loss of and obstructive remodeling of the pulmonary vascular bed is responsible for the rise in pulmonary arterial pressure and pulmonary vascular resistance (PVR), resulting in progressive right heart failure and functional decline [6]. While normal values of mean pulmonary artery pressure (mPAP) are considered to be 14 ± 3 mmHg, according to the European Society of Cardiology (ESC)/European Respiratory Society (ERS) common guidelines published in 2015, PH is defined as elevated mPAP more than 25 mmHg at rest, measured by right heart catheterization (RHC) [7]. In addition to invasively determining the mean pressure in the pulmonary artery, RHC identifies other hemodynamic parameters, which further divide PH into two groups: pre-capillary and post-capillary [7]. Pre-capillary PH is defined by pulmonary artery wedge pressure (PAWP) ≤15 mmHg and PVR ≥3 Wood Units (W.U.) [7]. Post-capillary PH is associated with high PAWP >15 mmHg, but PVR can vary: isolated postcapillary PH has PVR ≤3 W.U. and/or diastolic pressure gradient (DPG) <7 mmHg, whilst PVR ≥3 W.U. and/or DPG ≥7 mmHg show the association between post-capillary and pre-capillary PH [7].

The existence of pressure values in the “grey zone” (20–24 mmHg) was addressed by an update in the definition of PH proposed at the 6th World Symposium on Pulmonary Hypertension which includes a mPAP cut-off of 20 mmHg (not yet included in the guidelines) [8]. This dictates the necessity that patients with such mPAP and high risk of future development of PH (connective tissue diseases (CTD), hereditary PAH) are to be kept under close follow up [7].

## 2. Manuscript

### 2.1. SSc and PH

Clinical classification of PH includes five major groups, each one with its own subtypes [7]. PAH associated with CTD is part of the first group (subclass 1.4 along with the forms associated with HIV infection or portal hypertension). However, as a multisystemic disorder, SSc can also generate other types of PH: derived from left-heart disease, secondary to pulmonary causes or hypoxia, due to prothrombotic states, mainly if associated with antiphospholipid antibodies [4] (Table 1).

A comprehensive differential diagnosis is essential in order to correctly classify PH, as this is instrumental in establishing the appropriate therapeutic choices.

PAH prevalence in the population of SSc varies between 5–13% in multiple epidemiologic studies, including a recent systematic review; conversely 30% of all PAH causes are represented by CTD, mostly SSc [4,22,23]. DETECT, the first multicentric study using systematic RHC in patients with SSc at increased risk for PH, found PAH in 19% of patients, 70% of whom had lcSSc [24]. ILD and left-sided heart disease are also responsible for 1–1.4%, and 1–1.3% of all cases, respectively [24].

Cardiovascular disease caused by SSc is not limited solely to the elevation of blood pressure in the pulmonary circulation. Autoimmune disorders, in general, are associated with high cardiovascular risk, SSc not representing an exception in this regard [25]. In the setting of complex and multiple intense inflammatory chain reactions which these diseases generate, atherosclerosis is accelerated, myocardial fibrosis is described in both left ventricle (LV) and right ventricle (RV) with ensuing functional impairment, arrhythmogenesis is present, and conduction disturbances alongside pericarditis may occur [25]. Therefore, PH secondary to left heart disease is a likely occurrence.

Pulmonary veno-occlusive disease (PVOD) in SSc is very difficult to distinguish from PAH Group 1.4 because of a similar initial clinical picture consisting of exertion dyspnoea, fatigue and signs of heart failure [26]. However, oxygen saturation in the peripheral blood is usually lower and auscultatory pulmonary rales are more frequently found in PVOD and are associated with pulmonary oedema, especially after administration of pulmonary vasodilators.

A prothrombotic state can also be found in SSc, especially if antiphospholipid antibodies (aPL) are present, which was described in 10–24% of SSc patients in various cohorts [27,28], with a median percentage of aPL-positive patients of 14% in a recent systematic review [29].

Reported in a meta-analysis of 22 epidemiologic trials, the rate of survival in patients with SSc-PAH is 93%, 88% and 75% at 1, 2 and 3 years, respectively and at 3 years mortality rates reach 33%, representing, however, an improvement as compared to older cohorts due to the evolution of targeted therapies [30]. Moreover, compared to idiopathic pulmonary arterial hypertension (IPAH), SSc-PAH has worse outcomes because it does not respond as well to pulmonary vasodilators, despite a lower mPAP and similar reductions in cardiac index [4]. SSc-PAH is usually diagnosed several years after SSc discovery, and at the time of diagnosis most patients are classified in World Health Organization (WHO) functional class III/IV, with consecutive high mortality [5,31]. This leads to the necessity of instituting an early screening strategy in order to identify PH in patients with SSc, even asymptomatic ones, and to initiate specific treatment strategies.

### 2.2. Risk Factors for SSc-PAH

Several registries including PHAROS (Pulmonary Hypertension Assessment and Recognition of Outcomes in Scleroderma), REVEAL registry (Registry to Evaluate Early and Long-Term Pulmonary Arterial Hypertension Disease Management) and the French registry identified several risk factors for unfavorable clinical outcomes in patients with SSc-PAH: age over 60 years, male gender, dcSSc, WHO IV functional class, low diffusing capacity for carbon monoxide (DLCO) < 39%, systolic blood pressure (BP) ≤ 110 mmHg, distance at 6 min walking test (6MWT) < 165 m, mean pressure in the right atrium (RA) > 20 mmHg, and PVR > 32 W.U. [32,33,34,35] (Table 2).

Digital ulcers and osteolysis of the distal phalanges, even though not directly related to PAH, appear to predict development of groups 2 and 3 of PH [36]. Long-term evolution of Raynaud’s Phenomenon (RP) secondary to SSc also predisposes to PAH [22]. Presence of telangiectasia is a supplementary risk factor for the occurrence of PH [24].

Laboratory tests can help the clinician anticipate PH and its specific subtype: erythrocyte sedimentation rate (ESR) and elevated G immunoglobulins (IgG) can be an indicator for PH secondary to chronic lung disease or left-sided heart pathology [36]. Serum level of ACA also heightens the risk, while the presence of aPL can raise awareness for the occurrence of chronic thromboembolic pulmonary hypertension (CTEPH) [22,28].

Steen et al. demonstrated that a reduced DLCO is the best long-term predictor for the development of PH in SSc [37,38]. Most frequently, at the time of diagnosis, DLCO is significantly low (<50%) in the absence of coexistent ILD [37,38]. Steen et al. also found that, in comparison with those without PH, lcSSc cases and PH had DLCO reduced by 52%, 5 years before the detection of the systemic pathology [37]. Moreover, several studies showed that even 10 to 15 years before PH diagnosis, there was a marked decrease in DLCO value, while forced vital capacity (FVC) remained normal or near-normal [37].

### 2.3. Why Is Screening for SSc-PAH Important?

Pulmonary vasodilatory medication indicated for the treatment of PAH has significantly improved the prognosis for this category of patients [4]. Not only are there new and effective medications available, but early treatment was proven to generate better outcomes [39,40]. A multitude of clinical trials brought extra arguments for the utility of screening because of the beneficial effects exerted by different compounds: ARIES 1 and ARIES 2 (studies with endothelin antagonists) which evaluated patients with CTD and PAH demonstrated the increase in distance at the 6MWT and reduction of adverse clinical outcomes [41]. CTD associated PAH patients were also included in the SUPER and PHIRST trials showing the benefits of sildenafil and tadalafil respectively on symptoms relief [42,43]. PATENT-2 demonstrated that riociguat (guanylate cyclase stimulator) also improves distance at 6MWT and functional class [44]. However, maybe the most interesting in this regard is the GRIPHON trial (selexipag–prostacyclin analogue with oral administration) that published data according to which all-cause mortality rates were reduced by 40% in patients with PAH treated with this drug, especially early after diagnosis [45].

Because of pulmonary vasodilatory medication, survival rates of patients with SSc-PAH have improved from 12 months (1996) to 3 years in 60% of cases nowadays [46]. Most importantly, data from the ItinerAir SSc cohort demonstrated that systematic PAH detection programmes are able to identify those patients with milder disease and whose long-term survival prospects are better, compared with cases diagnosed during routine clinical practice [31,47]. Therefore, active PAH screening strategies are instrumental in improving the prognosis for SSc patients.

### 2.4. Methods and Screening Algorithms for PH in SSc

Screening is defined as the systematic testing of asymptomatic individuals in order to identify a certain pathology in the subclinical phase, or the testing of the mildly symptomatic to prevent the progression of the already manifesting disease [31]. Implementation of a screening program is justified when rapid detection can lead to the initiation of medication that can cure the patient or can modify the natural history of the disease [31].

#### 2.4.1. Clinical Examination

In contemporary practice, regular, complete and systematic clinical examination still remains fundamental. Unfortunately, excluding the typical features of SSc, in the early stages SSc-PAH is scarce in signs/symptoms. Clinical findings may be present only when the elevated pressure in the pulmonary artery causes the failure of the RV, which in turn can lead to loud cardiac 2 sound, jugular a wave, turgescent jugular veins, holo/mezotele-systolic heart murmur with maximum intensity in the tricuspid area, Graham-Steel murmur, and peripheral oedema, making clinical screening strategies inefficient [25,46].

Several SSc-related clinical features can also represent predictors for PAH development: digital ulcers, telangiectasia, and calcinosis [48].

#### 2.4.2. Nailfold Capillaroscopy

The progression of capillary loss as well as the evolution towards a severe (active/late) nailfold capillaroscopic pattern were found to be associated with incident SSc-PAH [49].

#### 2.4.3. 6MWT

An objective, accurate when performed correctly, reproducible and cheap method for detecting hypoxemia and worsening exertion levels in patients with PAH and SSc, the 6MWT was used as a marker of good outcomes in most clinical studies which evaluated the benefit of pulmonary vasodilatory medication [22].

Although it identifies poor exercise tolerance, as well as oxygen desaturation in the peripheral blood, it is not an adequate tool for PAH screening because it is neither sensible, nor specific [22]. Although the 6MWT distance is used as a risk marker in a multitude of screening algorithms, and small distances predict high rates of mortality, in patients with SSc it is not an appropriate outcome measurement when assessing for PAH, as it may be influenced by various other factors such as concomitant myopathy, myositis or arthritis [22,24].

#### 2.4.4. Laboratory Biomarkers

Extended SSc antibody status provides a better disease characterization and brings clues for possible SSc-related organ involvements, such as PAH. ACA are risk factors for SSc-PAH and patients should be carefully monitored in case of positivity [50]. Moreover, the inflammatory status might reflect the worsening of the disease. C-reactive protein (CRP) levels higher than 8 mg/L were identified as an independent predictor for PAH poor prognosis [35].

Biomarkers such as brain natriuretic peptide (BNP) and its N-terminal fragment (NTproBNP) are objective tools for detecting cardiac dysfunction, irrespective of etiology. However, the circulating levels of NTproBNP do not vary consistently across all types of PAH: in CTD associated PAH their levels are high compared to IPAH, despite milder hemodynamic disturbances [31].

Williams et al. reported a cut-off value of 395 pg/mL for NTproBNP, with 55.9% sensitivity and 95.1% specificity for detecting SSc associated PAH [51]. Moreover, in a large cohort of patients, using 125 ng/L concentration as a threshold value, NTproBNP reliably and independently predicted 3-year mortality, with a sensitivity of 78.1% and a negative predictive value (NPV) of 97.6%, respectively [52]. More recent studies also included cardiac troponin as a prognostic biomarker, probably by reflecting myocardial injury due to both SSc-related myocardial fibrosis, as well as PH-related myocardial stress [53].

Red cell distribution width (RDW) is a quantitative measure of circulating erythrocytes size. In a prospective clinical trial consisting of 145 patients with SSc-PAH, Zhao et al. demonstrated the presence of an independent association between RDW and PH both in lcSSc and dcSSc [54]. RDW is inversely proportional to DLCO and its elevation can predict the worsening of cardio-pulmonary function in patients with SSc [54].

Uric acid levels were shown to be elevated in chronic hypoxic states like obstructive pulmonary disease or chronic heart failure. In SSc, determination of uric acid level together with BP monitoring are recommended for the early detection of scleroderma crisis. Moreover, serum levels of uric acid are considered to be a biomarker for the severity of PH related ventricular dysfunction [55].

#### 2.4.5. Echocardiography

Annual echocardiography of SSc patients remains the cornerstone of PH screening. By estimating pressures and measuring cavitary dimensions, volumes, systolic and diastolic function parameters, echocardiography is able to detect all possible cardiovascular complications of the disease. Moreover, it is the most efficient screening method because it is non-invasive, cost-efficient and virtually readily available everywhere [31,56]. Thus, European and American clinical practice guidelines recommend that patients with SSc are to be evaluated by echocardiography even if they are asymptomatic [25].

Twenty-eight echocardiographic values have been ascertained in the DETECT study (i.e., RA and RV areas, RA diameter, tricuspid regurgitation (TR) velocity, tricuspid annular plane systolic excursion (TAPSE)), but only the velocity of the TR and areas of RA and RV demonstrated utility in detecting PH [57].

Several echocardiographic abnormalities, when present, might indicate PH and thus can be an incentive for referring the patient to RHC: systolic pulmonary artery pressure (sPAP) ≥ 40 mmHg, TR velocity (TRV) > 2.8 m/sec, RA dimensions >53 mm and RV dimensions > 35 mm [25]. If sPAP values < 36 mmHg and TRV < 2.8 m/s are not accompanied by other echocardiographic signs suggestive of PH, then its presence is unlikely [24]. On the other hand, velocity of regurgitation > 3.4 m/s and sPAP estimated over 50 mmHg are strong indicators of high likelihood of PH [24]. Compared to RHC, echocardiography has a 39–100% sensitivity and 42–97% specificity for detecting PH [24]. Beyond high pressure estimates and right cavities’ dilation, several echocardiographic features can coexist in PH, such as interventricular septum flattening, dilation of the inferior cava, and short pulmonary artery flow acceleration time [7]. Still, as a singular method used for early detection of PH, echocardiography can sometimes be inaccurate in evaluating PAP, over or under-estimating it [4]. Typically, it over-estimates sPAP with more than 10 mmHg which makes it a low efficiency screening method in mild or asymptomatic patients [24]; under-estimation is also possible especially if incomplete TR envelopes are obtained or RV dysfunction coexists. Operator experience is of the utmost importance, PAP value being unobtainable in 20–39% of cases [31].

Therefore, present guidelines suggest a combination of echocardiographic parameters in order to raise suspicion of PH, especially in the grey zone of TRV between 2.8–3.4 m/s [7] (Table 3 and Table 4).

Other less used parameters in clinical practice for this aim, such as the left atrium (LA) volume and the RV myocardial performance index, are independent predictors of PAH in SSc patients [25]. Pulmonary pulse wave transit time (PPTt) was suggested by Wibmer et al. to be a surrogate marker for the hemodynamic disturbances which the vascular bed suffers in PH [58]. It was demonstrated that PPTt is low in patients with SSc compared to healthy subjects and that it can be considered an early result of inflammatory alterations caused by the systemic disease in the small pulmonary vessels [59]. Using speckle tracking echocardiography, Hekimsoy et al. demonstrated that apical RV free wall strain was lower in patients with SSc-PAH compared to those without PH (−14.6 ± 5.9 vs. −22.2 ± 7.5, *p* = 0.03) [60]. This led to the conclusion that in scleroderma the apical RV free wall strain has high specificity for PAH [60].

However, due to high intra- or inter-observer variability and lack of clear cut-off limits, these observations cannot yet be incorporated into screening strategies.

#### 2.4.6. Exercise Testing

While detecting PH with resting echocardiography remains the essential clinical tool, several studies suggested that in patients with SSc, an even earlier change would be the abnormal increase in pulmonary pressures with exercise. A study of 25 cases with normal resting PAP demonstrated reduced RV contractile reserve with exercise, suggesting that subclinical RV dysfunction during physical stress might be a surrogate for early pulmonary vascular disease [61]. Moreover, increments in measured sPAP on exercise echocardiography can be followed over time, indicating the progression of pulmonary vascular disease before overt PAH is diagnosed [62].

Therefore, exercise echocardiography can be a helpful tool in detecting early changes. However, data regarding its use is currently limited and more studies are needed to evaluate the usefulness of this non-invasive method.

Another potentially promising modality for screening of early SSc-PAH is cardiopulmonary exercise testing, as demonstrated by Dumitrescu et al., which found a peak rate of oxygen consumption (peak VO_2_) >18.7 mL/kg/min and nadir minute ventilation to CO_2_ production ratio (nadir VE/VCO_2_) >45.5 to be the most accurate parameters for excluding pathologically elevated pulmonary artery pressures at diagnosis [63].

#### 2.4.7. Pulmonary Function Tests

Pulmonary function tests (PFT) are part of the standard periodic evaluation of the SSc patient due to ILD complications, but are also used for the early detection of PH. It is mandatory to do PFTs once a year or earlier in case the clinical picture suffers changes [4]. Complete evaluation of pulmonary function must be achieved at patient’s first visit because it brings valuable important information regarding two of the most prevalent types of pulmonary disease in this population (PAH, ILD), by: lung volumes, spirometry, DLCO [22].

Steen et al. were the first to demonstrate that patients with PAH and lcSSc have significantly lower mean DLCO, up to almost half of the predicted value, 5 years before diagnosis [37]. DLCO < 60% of the predicted value increases the risk of PAH by more than five times [37]. Because of this, patients with scleroderma and low DLCO are considered to be at high risk for future development of this medical condition [31]. ItinerAir data have shown that at a DLCO > 60% of the predicted values, specificity for excluding PAH is high, a fact that afterwards became the base for including DLCO in any PH screening programs, setting the threshold at <60% predicted [64]. Not only the absolute values, but also progressive decline can be a sign for SSc-PAH.

Moreover, restrictive spiro-metric pattern (reduced total lung capacity (TLC), reduced FVC) is a strong indicator for ILD and the ratio of FVC/DLCO > 1.6 is an independent, strong predictor for PAH [65].

### 2.5. Screening Algorithms

Having all these tools for investigating the SSc patients in PAH screening, the further question is how to best combine and use them in a cost-effective way, with the best positive predicted value (PPV) and NPV. Several such algorithms were proposed by recent guidelines and studies, detailed below. The essential common idea remains the indication to actively screen patients with SSc or mixed connective tissue disease (MCTD) for PH, without waiting for clinical signs to be the first diagnostic element. Nevertheless, additional studies are needed to determine the most cost-effective strategy for implementing an optimal duration and screening period [66,67] (Table 5).

#### 2.5.1. 2015 ESC/ERS Guidelines for the Diagnosis and Treatment of Pulmonary Hypertension

The most frequently used recommendations for PH screening arise from the 2015 ESC/ERS guidelines and are based on the presence of symptoms and TR [7,70]. Still, there are certain limitations to this approach since in the early phases of the disease symptoms might be absent and tricuspid insufficiency is missing in 20%–39% of cases, facts that paved the way for the development of new algorithms [70].

In the current guidelines, positive screening implies TRV > 3.4 m/s, or between 2.8 and maximum 3.4 m/s, with symptoms (dyspnea, syncope/presyncope, peripheral oedema) and a supplementary echocardiographic parameter suggestive for elevated pulmonary artery pressure (Table 2 and Table 3) [70].

#### 2.5.2. DETECT Algorithm

**Figure 1:**
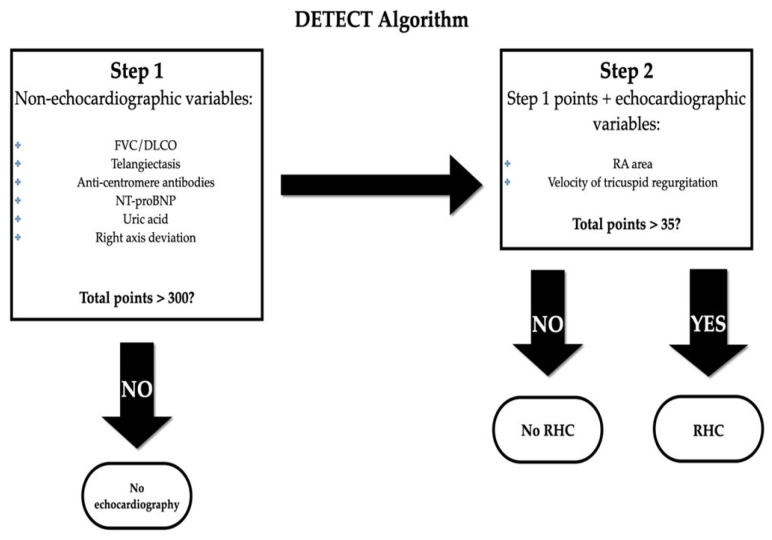
DETECT Algorithm. Modified after [57]. Legend: FVC forced vital capacity; DLCO diffusing capacity for carbon monoxide; NTproBNP, N-terminal pro-brain natriuretic peptide; RA, right atrium.

The DETECT study included 464 patients with SSc at increased risk for future development of PAH (more than 3 years of disease duration since the first non-Raynaud symptom and a DLCO < 60%) and performed systematic non-invasive testing and RHC [57]. After multivariable analysis, a two-step screening model was proposed, making use of echocardiography only in those cases which were truly at high risk for PAH [57]. Figure 1 illustrates the DETECT screening algorithm.

It is composed using 6 non-echocardiographic parameters: FVC/DLCO, telangiectasias, ACA, NTproBNP, uric acid, right axis deviation on electrocardiography (ECG) [4]. If the obtained score (calculated via an online website: detect-pah.com, last accessed on Mai 20, 2021) is higher than 300, progression towards echocardiography occurs, and based on this result a recommendation for invasive hemodynamic assessment is issued or not [4]. The sensitivity of the DETECT algorithm is 96%, with a high NPV of 98% for the early detection of PAH, but the clinical trial was centered around a scleroderma population at high risk for PH (DLCO <60%) and it did not include patients with significant ILD [4]. Specificity was lower (48%), and up to 62% of cases were referred to RHC, leading to many false positives undergoing further invasive assessment.

Yanjie Hao et al. compared the accurate predictability of DETECT 2013 with 2009 ESC/ERS guidelines and concluded that the sensitivity and NPV of DETECT are superior [70]. The advantage is represented by the fact that the former includes more variables compared to the latter and it does not rely strictly on symptoms/TR, so it can be applied also to those patients who do not have tricuspid insufficiency [70]. Moreover, DETECT is not validated for cases with DLCO > 60%.

#### 2.5.3. ASIG Algorithm

The Australian Scleroderma Interest Group (ASIG) algorithm uses a different set of parameters, combining NTproBNP (with a cutoff of 210 pg/mL) and PFT (assessment of FVC/DLCO with a cutoff of 1.8). If any of these two parameters is found to be abnormal, the patient is further referred for echocardiography and possibly RHC [71].

**Figure 2:**
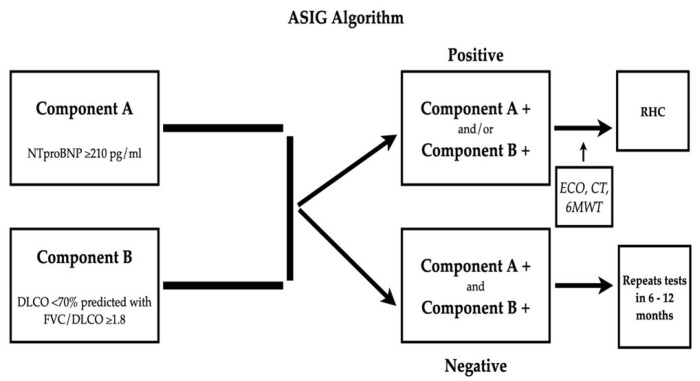
ASIG Algorithm. Modified after [70]. Legend: FVC forced vital capacity; DLCO diffusing capacity for carbon monoxide; NTproBNP, N-terminal pro-brain natriuretic peptide.

ASIG was published shortly after DETECT and the higher specificity (54.5%), NPV (92.3%) and PPV (61.5%) compared to the ESC/ERS guidelines have been confirmed in a validation cohort [70]. Contrary to DETECT, ASIG has higher specificity, meaning that the rate of cases sent to RHC was lower at 12%, without missing any scenario of PH, proving more cost-effective [70]. Figure 2 presents the ASIG algorithm.

Positive screening is considered if any of the two components (A or B) or both meet the criteria and, in order for it to be negative, none of the criteria must be met [70].

#### 2.5.4. Other Screening Algorithms

Table 6 presents three other screening strategies for estimating the probability of a patient developing PH. However, less data regarding their sensibility and predictive values are available.

### 2.6. Screening Strategies–Are They All the Same?

In a screening program, the most important performance measures are sensitivity and NPV, as their high values announce the high ability of a strategy to capture all affected patients.

A study by Hao et al. compared the three most utilized screening algorithms, DETECT, ASIG and ESC/ERS 2009 guidelines, in 73 SSc patients [70]. They demonstrated that both DETECT and ASIG had higher sensitivity and NPV compared to the ESC/ERS algorithm. Being multiparametric, they outperformed the echocardiographic algorithm by also being applicable in patients without a measurable tricuspid gradient. The DETECT and ASIG algorithms seemed comparable (sensitivity 97.4% and 95.8%, respectively; NPV 92.3% for both). The ASIG strategy might appear as less costly as it only involves two parameters.

## 3. Conclusions

PH in SSc is the result of several phenotypes, part of groups 1, 2, 3 or 4 in the PH classification, and even a combination of these. Current investigations do not always discriminate well between different forms of SSc associated PH, making management choices more difficult.

There is a clear consensus on the recommendation that all patients with SSc or MTCD with SSc-features should undergo active and periodic PH screening, because early diagnosis of this complication allows timely vasodilator therapy and better prognosis. Therefore, a local screening algorithm should be established; based on this, all patients with SSc with positive non-invasive testing for PH must be referred to an expert center, in order to undergo RHC and further therapeutic decisions.

## Figures and Tables

**Table 1:** Possible causes of PH in SSc. Legend: PAH–pulmonary arterial hypertension, PVOD–pulmonary veno-occlusive disease, ILD–interstitial lung disease, DLCO–diffusing capacity for carbon monoxide, CTEPH–chronic thromboembolic pulmonary hypertension.

Group	PH Type	Observations	Ref
Group 1.4	Pulmonary arterial hypertension	Remodelling and constriction of the pulmonary arteries and arterioles, with progressive increase of the pulmonary vascular resistance and right heart failure	[4,9,10,11]
Group 1	Pulmonary veno-occlusive disease	More pronounced venous/capillary involvement; associated with poor prognosis, limited response to PAH therapy which increases risk of pulmonary oedema	[12,13,14,15]
Group 2	PH secondary to left heart disease	Myocardial fibrosis with diastolic dysfunction, possible late systolic dysfunction	[16]
Group 3	PH secondary to lung disease/hypoxia	SSc with ILD can lead to hypoxia depending on the severity of fibrosis and DLCO depreciation	[16,17,18,19]
Group 4	Chronic thromboembolic pulmonary hypertension	Prothrombotic state especially due to antiphospholipid antibodies presence	[20,21]

**Table 2:** Risk factors for PH development in SSc. Legend: WHO–World Health Organization, ESR-erythrocyte sedimentation rate, 6MWT–six-minute walking test, DLCO-diffusing capacity for carbon monoxide, RHC-right heart catheterization, RA–right atrium, SBP–systolic blood pressure, IgG–G immunoglobulins, PVR–pulmonary vascular resistance, W.U.–Wood Units, ACA–anti-centromere antibodies, RP–Raynaud’s Phenomenon.

Demographic Characteristics	>60 years old [32,33] Male gender [32,33]
Clinical Factors	WHO functional class IV [33] SBP ≤ 110 mmHg [33] Digital ulcers [36] Osteolysis of the distal phalanges [36] Long term evolution of RP and long disease duration [22] Telangiectasia [24]
Laboratory Biomarkers	Elevated ESR [36] Elevated IgG [36] ACA [22] Antiphospholipid antibodies [28]
6MWT	<165 m [32,33]
Pulmonary Function Testing	DLCO < 39% [32]
Right Heart Catheterization	Mean RA pressure > 20 mmHg [33] PVR > 32 Wood Units [33]

**Table 3:** Echocardiographic assessment of PH probability. Modified after [7]. Legend: TR–tricuspid regurgitation, PH–pulmonary hypertension. See Table 4 for supplementary echocardiographic signs.

Peak TR Velocity (m/s)	Presence of Other Echocardiographic PH Signs	Echocardiographic Probability of PH
≤2.8 or not measurable	No	Low
≤2.8 or not measurable	Yes	Intermediate
2.9–3.4	No	
2.9–3.4	Yes	High
>3.4	Not required	

**Table 4:** Supplementary echocardiographic signs suggestive of PH. Modified after [7]. Legend: RV–right ventricle, LV–left ventricle, IVC–inferior vena cava, RA–right atrium.

A: The Ventricles	B: Pulmonary Artery	C: IVC and RA
RV/LV basal diameter ratio > 1	RV outflow Doppler acceleration time <105 ms and/or midsystolic notching	IVC > 21 mm with decreased inspiratory collapse
Flattening of the interventricular septum (LV eccentricity index > 1.1 in systole and/or diastole)	Early diastolic pulmonary regurgitation velocity > 2.2 m/s	RA area (end-systole) > 18 cm^2^
	Pulmonary artery diameter > 25 mm	

**Table 5:** Recommendations for the evaluation of patients with PAH and connective tissue disease. Modified after [7]. Legend: DLCO, diffusing capacity for carbon monoxide; IPAH, idiopathic pulmonary arterial hypertension; PAH, pulmonary arterial hypertension; RHC, right heart catheterization; SSc, systemic sclerosis.

Recommendations	Class	Level of Evidence	Ref
In patients with PAH associated with connective tissue disorder, the same treatment algorithm as for patients with IPAH is recommended	I	C	[64]
Resting echocardiography is recommended as a screening test in asymptomatic patients with SSc, followed by annual screening with echocardiography, DLCO and biomarkers	I	C	[64]
RHC is recommended in all cases of suspected PAH associated with connective tissue disease	I	C	[57,64]
Oral anticoagulation may be considered on an individual basis and in the presence of trombophilic predisposition	IIa	C	[68,69]

**Table 6:** Other PH screening methods. Modified after [72,73,74].

	Di (distance) BO (Borg) SA (SpO_2_)–DIBOSA [72]	Cochin [73]	Telangiectasis [74]
Parameters	6MWT distance Dyspnea–Borg Scale O_2_ saturation at 6 min	Age FVC DLCO/Alveolar volume	Telangiectasis at multiple anatomical sites
Interpretation	<360 m–1; ≥360 m–0 >2–1; ≤2–0 <95%-1; ≥95%-0	High Cochin score	0–no telangiectasis 1–between 1 and 10 2–more than 10
Result	0–no risk for PH 1–30% risk 2–48% risk 3–86% risk	35 times higher risk to develop PAH compared to the general population	For every 10 points, mPAP had a mean elevation of 10.9 mmHg

## Data Availability

Not applicable.
